# Flow Matching for Count Data

**Published:** 2026-05-08

**Authors:** Ganchao Wei, John Pearson

**Affiliations:** Department of Neurobiology, Department of Statistical Science, Duke University, Durham, NC, USA; Department of Neurobiology, Department of Electrical and Computer Engineering, Duke University, Durham, NC, USA

## Abstract

High-dimensional count data arise in applications such as single-cell RNA sequencing and neural spike trains, where mapping between distributions across successive batches or time points form critical components of data analysis. The recent success of diffusion- and flow-based deep generative models for images, video, and text motivates extending these ideas to count-valued settings, but many existing methods either treat each count as a categorical state or transform counts into a continuous space, neither of which is natural or efficient when the count range is large. We propose count-FM, a flow-matching framework for count data based on a continuous-time birth-death process with local unit jumps. Count-FM learns marginal transitions efficiently in count space through simulation-free training of conditional transition rates, allowing transport between arbitrary count-distributed source and target populations. In simulation, count-FM achieves better sample quality than representative baselines while using substantially fewer parameters. We further apply count-FM to scRNA-seq and neural spike-train data for unconditional generation, transport, and conditional generation. Across these tasks, count-FM yields improved sample quality, greater modeling efficiency, and interpretable transport paths.

## 1 Introduction

High-dimensional count data arise in many scientific applications, including key biological data types such as single-cell RNA sequencing and neural spike trains. However, their discreteness, sparsity, and complex correlation structure make flexible joint modeling difficult, especially in high dimensions. Deep generative models provide a powerful framework for addressing this problem, with major developments including variational autoencoders [Kingma and Welling, 2022], generative adversarial networks [Goodfellow et al., 2014], normalizing flows [Kingma and Dhariwal, 2018], diffusion models [Ho et al., 2020, Song et al., 2021], and flow matching [Lipman et al., 2023]. In particular, diffusion- and flow-based methods have recently attracted substantial attention because of their strong empirical performance, flexible path-based formulations, and efficient training procedures [Ho et al., 2020, Song et al., 2021, Lipman et al., 2023]. This progress has motivated growing interest in extending these generative frameworks from continuous domains to discrete data.

For modeling discrete data, recent approaches include diffusion models on categorical state spaces [Austin et al., 2021, Lou et al., 2024], continuous-time Markov chain formulations [Campbell et al., 2022, 2024], and flow-based constructions on discrete domains [Stark et al., 2024, Gat et al., 2024]. These methods are primarily designed for categorical variables such as tokens or labels. When they are adapted to multivariate count data, one common choice is to represent each variable using a categorical distribution over all values, up to a maximum count. This treats adjacent counts as unrelated categories and requires that the output dimension scales with the count range. Another approach is to map the data into a continuous space through dequantization or continuous latent representations [Ho et al., 2019, Hoogeboom et al., 2021], but this replaces probability mass on counts by a continuous density and can blur the underlying discrete structure [Hoogeboom et al., 2019, Luo et al., 2021]. Count-specific jump models such as Poisson-JUMP [Chen and Zhou, 2023] provide another count-valued alternative, but they are not designed for transport between arbitrary count distributions.

To address these challenges while retaining the efficient training and generation of flow matching, we propose count-FM, a flow-matching framework for count data based on a continuous-time birth-death process with local unit jumps. We model transport directly in count space through coordinate-wise local births and deaths, so that transitions respect the geometry of counts and remain count-valued along the path. The conditional binomial bridge yields an efficient training objective and enables tractable training of the marginal transition rates. The model is also parameter-efficient, since it predicts only local birth and death rates rather than a full categorical distribution over all count levels. This makes count-FM well suited to high-dimensional count data with large count ranges.

We first validate count-FM on a two-dimensional simulation before applying it to single-cell RNA-seq for unconditional generation and transport, followed by conditional generation of brain data, multiregion hippocampal and entorhinal spike trains. Our main contributions are as follows:

- We propose count-FM, a flow-matching framework for count data based on a continuous-time birth-death process with local unit jumps, so that transport is modeled directly in count space and intermediate states remain count-valued along the path.
- We develop a tractable training scheme for marginal transition rates through a conditional binomial bridge while retaining the efficient training and generation of flow matching.
- We obtain a parameter-efficient formulation that predicts only local birth and death rates rather than a full categorical distribution over all count levels, making it well suited to high-dimensional count data with large count ranges.

## 2 Method

In this paper, we consider a pair of counting distributions on d variables, $N_{0}^{d}=\{0,1,2,\ldots {\}}^{d}$, where $x_{0}\sim p_{0}$ is a source sample and $x_{1}\sim p_{1}$ is a target sample. We model the transition between these two distributions as a continuous-time Markov jump process (CTMC) [Campbell et al., 2022, 2024] with local unit births and deaths. Let $X_{t}\in N_{0}^{d}$ denote the count vector at time t, and let $e_{i}\in R^{d}$ denote the ith standard basis vector, that is, the vector whose ith entry is 1 and all other entries are 0. Over a small time interval h>0, the process evolves according to

$$P\left(X_{t+h}=x+e_{i}|X_{t}=x\right)=h\;\lambda_{t,i}(x)+o(h),$$

$$P\left(X_{t+h}=x-e_{i}|X_{t}=x\right)=h\;\mu_{t,i}(x)+o(h),$$

and the probability of staying at x is

$$P\left(X_{t+h}=x|X_{t}=x\right)=1-h\sum_{i=1}^{d}\left(\lambda_{t,i}(x)+\mu_{t,i}(x)\right)+o(h).$$

Death is disallowed when $x^{\left(i\right)}=0$. The goal is to learn the time-dependent birth rates $\lambda_{t}(x)$ and death rates $\mu_{t}(x)$.

One benefit of this local birth-death parameterization is parameter efficiency for count-valued data. Other discrete diffusion- or flow-based models for count data represent each coordinate as a categorical variable over all count levels up to a maximum count, so the total output dimension is $\sum_{i=1}^{d}\left(C_{i}+1\right)$, where $C_{i}$ denotes the maximum count for coordinate i. By contrast, count-FM parameterizes each coordinate using only a local birth and death rate (±1), so its state-dependent output dimension is always 2d, regardless of maximum counts. This is more important in high-count and high-dimensional settings, where categorical-state parameterizations grow increasingly expensive. Empirically, in both the simulation and scRNA experiments, count-FM uses substantially fewer trainable parameters than the competing categorical-state baselines (Tables 1 and 2).

### 2.1 Conditional count bridge and training objective

Given a pair of endpoints $\left(x_{0},x_{1}\right)$, we define the conditional bridge coordinatewise. For each coordinate i,

(1) $$X_{t}^{\left(i\right)}=x_{0}^{\left(i\right)}+\text{sgn}\left(x_{1}^{\left(i\right)}-x_{0}^{\left(i\right)}\right)B_{t}^{\left(i\right)},\;B_{t}^{\left(i\right)}\sim \text{Binomial}\left(\left|x_{1}^{\left(i\right)}-x_{0}^{\left(i\right)}\right|,t\right),$$

independently across coordinates given ${(x}_{0},x_{1})$, where sgn is the sign function. This bridge defines a conditional probability path $p_{t}\left(x|x_{0},x_{1}\right)$ whose coordinate-wise mean moves linearly from $x_{0}$ to $x_{1}$, with local count changes.

For a fixed coordinate i, let $x=X_{t}^{\left(i\right)}$ and write $p_{t}^{\left(i\right)}\left(x|x_{0},x_{1}\right)$ for the corresponding one-dimensional bridge marginal. Then mass preservation (equivalently, the one-dimensional Kolmogorov forward equation for a local CTMC [Holderrieth et al., 2025]) gives

(2) $$\partial_{t}p_{t}^{\left(i\right)}\left(x|x_{0},x_{1}\right)=\lambda_{t,i}\left(x-1|x_{0},x_{1}\right)p_{t}^{\left(i\right)}\left(x-1|x_{0},x_{1}\right)+\mu_{t,i}\left(x+1|x_{0},x_{1}\right)p_{t}^{\left(i\right)}\left(x+1|x_{0},x_{1}\right)\;-\left(\lambda_{t,i}\left(x|x_{0},x_{1}\right)+\mu_{t,i}\left(x|x_{0},x_{1}\right)\right)p_{t}^{\left(i\right)}\left(x|x_{0},x_{1}\right).$$

This equation expresses local mass balance, where the change in mass equals influx minus outflux. Requiring the conditional rates to satisfy it ensures that the CTMC generates the conditional path $p_{t}\left(\cdot |x_{0},x_{1}\right)$. Substituting the conditional binomial bridge (1) into (2) then yields,

$$\lambda_{t,i}\left(x|x_{0},x_{1}\right)=\frac{{\left(x_{1}^{\left(i\right)}-x^{\left(i\right)}\right)}_{+}}{1-t},\;\mu_{t,i}\left(x|x_{0},x_{1}\right)=\frac{{\left(x^{\left(i\right)}-x_{1}^{\left(i\right)}\right)}_{+}}{1-t},$$

where $(\cdot {)}_{+}$ indicates positive rectification. In practice, we replace 1-t by $1-t+\varepsilon_{t}$ to avoid numerical blow-up near t=1. See Appendix A.1 for the detailed derivation. The associated marginal transition rates ${(\lambda }_{t},\mu_{t})$ are obtained by averaging over endpoint pairs ${(x}_{0},x_{1})$ ~π, and they generate the marginal probability path $p_{t}(x)=E_{\left(x_{0},x_{1}\right)\sim \pi }\left[p_{t}\left(x|x_{0},x_{1}\right)\right]$. (Holderrieth et al. [2025], prop. 1) As shown in generator matching framework (Holderrieth et al. [2025], prop. 2), training with these conditional transition rates can be viewed as a equivalent to learning the marginal transition rates $\left(\lambda_{t},\mu_{t}\right)$, in the sense that the induced population objective has the same gradients with respect to the model parameters. Thus, by training on the identifiable conditional binomial bridge, learning the marginal rates becomes tractable.

To ensure zero death rate at the boundary x=0, we parameterize the model by nonnegative birth rates $\lambda_{\theta }(x,t)$ and death rates $\mu_{\theta }(x,t)=x⊙\beta_{\theta }(x,t)$, where the death coefficients $\beta_{\theta }(x,t)$ are nonnegative. In the following experiments, birth and death rates are modeled using a single neural network with separate outputs. We train the model by minimizing a path-space KL induced by the conditional count bridge. Let $X_{\left(0,1\right]}≔\left\{X_{s}:0<s\leq 1\right\}$. Then, up to a constant c independent of θ,

(3) $${ℒ}_{\text{train}}(\theta )=E_{\left(x_{0},x_{1}\right)\sim \pi }\left[\text{KL}\left(p\left(X_{\left(0,1\right]}|X_{0}=x_{0},X_{1}=x_{1}\right)\|p_{\theta }\left(X_{\left(0,1\right]}|X_{0}=x_{0}\right)\right)\right]+c.$$

For the local birth-death process, this path-space KL decomposes into local one-step KL terms. Taking the infinitesimal limit gives a generalized KL divergence between the conditional bridge rates and the model jump rates. Dropping terms independent of θ gives the practical training objective

(4) $${ℒ}_{\text{train}}(\theta )=E_{\begin{array}{c} \left(x_{0},x_{1}\right)\sim \pi ,\\ \underset{x\sim p_{t}\left(\cdot |x_{0},x_{1}\right)}{t\sim \text{Unif,}} \end{array}}\left[\sum_{i=1}^{d}\ell \left(\lambda_{t,i}\left(x|x_{0},x_{1}\right),\lambda_{\theta ,i}(x,t)\right)+\sum_{i=1}^{d}\ell \left(\mu_{t,i}\left(x|x_{0},x_{1}\right),\mu_{\theta ,i}(x,t)\right)\right],$$

where ℓ(u,v)=v-ulogv and $\pi \left(x_{0},x_{1}\right)$ is the coupling distribution between endpoints. The logv is replaced by $\text{log}\left(v+\varepsilon_{\ell }\right)$ for numerical stability in practice. This is analogous to the data-augmentation view of continuous diffusion objectives [Kingma and Gao, 2023], where local training losses can be interpreted as optimizing a global probabilistic objective over the augmented process. The local KL derivation and the path-space KL interpretation are given in Appendices A. 2 and A.3, respectively.

### 2.2 Sample generation

After training, we generate samples by simulating the learned birth-death process forward from $t=\varepsilon_{t}$ to $t=1-\varepsilon_{t}$, where, again, $\varepsilon_{t}$ is introduced to avoid the singularity at t=1. The initial state is sampled from the source distribution $p_{0}$, which can be either a simple count-valued distribution (for unconditional generation) or the observed source population. Furthermore, we use a first-order local-jump discretization: With K steps and step size $\Delta =\left(1-2\varepsilon_{t}\right)/K$, define the total jump rate for coordinate i at state x and time t as

$$r_{i}\left(x,t\right)=\lambda_{\theta ,i}\left(x,t\right)+\mu_{\theta ,i}\left(x,t\right).$$

For each coordinate i, we sample one of three outcomes,

$$p_{i}^{\text{stay}}=\text{exp}\left(-r_{i}\Delta \right),\;p_{i}^{\text{birth}}=\left(1-\text{exp}\left(-r_{i}\Delta \right)\right)\frac{\lambda_{\theta ,i}}{r_{i}+\varepsilon_{r}},\;p_{i}^{\text{death}}=\left(1-\text{exp}\left(-r_{i}\Delta \right)\right)\frac{\mu_{\theta ,i}}{r_{i}+\varepsilon_{r}},$$

where $\varepsilon_{r}$ is for numerical stability when $r_{i}=0$. The next state is obtained by applying the sampled unit update in each coordinate. Applying this update sequentially from the initial state to t=1 yields an approximate sample trajectory.

### 2.3 Endpoint coupling

The endpoint coupling $\pi \left(x_{0},x_{1}\right)$ determines how source and target samples are paired during training and thus influences the learned marginal transition path. Here, we either consider independent coupling, $\pi_{\text{ind}}\left(x_{0},x_{1}\right)=p_{0}\left(x_{0}\right)p_{1}\left(x_{1}\right)$, (**count-FM**) or a minibatch optimal-transport (OT) coupling (**count-FM-OT**), similar to OT-CFM [Tong et al., 2024]. For the latter, we draw source and target minibatches ${\left\{x_{0,b}\right\}}_{b=1}^{B}$ and ${\left\{x_{1,b}\right\}}_{b=1}^{B}$. These define empirical measures ${\overset{ˆ}{p}}_{0}^{B}=\frac{1}{B}\sum_{b=1}^{B}\delta_{x_{0,b}}$ and ${\overset{ˆ}{p}}_{1}^{B}=\frac{1}{B}\sum_{b=1}^{B}\delta_{x_{1,b}}$, both with uniform weights $u_{B}=\frac{1}{B}1_{B}$. We then compute the empirical OT coupling

$$\Gamma^{⋆}=\text{arg}\underset{\Gamma \in \Pi \left(u_{B},u_{B}\right)}{\text{min}}\sum_{b=1}^{B}\sum_{b^{\prime }=1}^{B}\Gamma_{bb^{\prime }}c\left(x_{0,b},x_{1,b^{\prime }}\right),$$

where $\Pi \left(u_{B},u_{B}\right)$ denotes the set of joint distributions on the two minibatches with marginals $u_{B}$. We sample endpoint pairs $\left(x_{0},x_{1}\right)$ from $\Gamma^{⋆}$ and then use the same conditional bridge construction and training objective as in Section 2.1. This minibatch OT step can be viewed as an empirical approximation to a population coupling between $p_{0}$ and $p_{1}$. We use the symmetric Poisson cost

$$c\left(x,y\right)=\sum_{i=1}^{d}\left[x_{i}\;\text{log}\frac{x_{i}+\varepsilon_{c}}{y_{i}+\varepsilon_{c}}+y_{i}\;\text{log}\frac{y_{i}+\varepsilon_{c}}{x_{i}+\varepsilon_{c}}\right],$$

where $\varepsilon_{c}>0$ is a small constant for numerical stability. This is the symmetrized generalized KL divergence between count vectors.

In the experiments, we report both count-FM and count-FM-OT for unconditional generation. The OT coupling changes the endpoint coupling, and hence modifies the geometry of the learned marginal transport path. In practice, OT coupling tends to induce transitions with lower curvature, which is beneficial for interpretation. It can also improve sampling efficiency, in the sense that under the same discretization scheme, similar sample quality may be achieved with fewer function evaluations (NFEs) or less wall-clock time. We verify this empirically in Appendix Figure 5, where the OT-coupled version generally reaches a given quality level more quickly than the independently coupled version.

### 2.4 Conditional generation

For conditional generation, we augment the model with covariates y and use conditional rates $\lambda_{\theta }(x,t,y)$ and $\mu_{\theta }(x,t,y)$. We train the conditional model with classifier-free guidance (CFG) [Ho and Salimans, 2021]: During training, conditioning variables are randomly dropped and replaced by learned null embeddings, allowing a single network to learn both conditional and unconditional rates. This avoids training a separate auxiliary classifier and provides a simple way to control the strength of conditioning at sampling time through the guidance scale. At sampling time, we combine the conditional and unconditional rates as

$$\lambda_{\theta }^{\text{cfg}}=\lambda_{\theta }^{\text{uncond}}+w\left(\lambda_{\theta }^{\text{cond}}-\lambda_{\theta }^{\text{uncond}}\right),\;\mu_{\theta }^{\text{cfg}}=\mu_{\theta }^{\text{uncond}}+w\left(\mu_{\theta }^{\text{cond}}-\mu_{\theta }^{\text{uncond}}\right),$$

where w≥0 is the guidance scale. Here, w=0 recovers unconditional generation, 0<w<1 interpolates between unconditional and conditional generation, and w=1 gives the standard conditional model. Values w>1 strengthen the influence of the conditioning variables beyond the standard conditional model, which can improve condition alignment and sharpen condition-specific structure. At the same time, overly large w may distort the marginal distribution and reduce diversity.

## 3 Simulation

We begin with a two-dimensional example simulation designed to evaluate both sample quality and intermediate transport behavior. The target distribution is an equal-weight mixture of two Gamma-Poisson components with modes near (60, 5) and (60, 40). For a fair comparison, all models except Poisson-JUMP [Chen and Zhou, 2023] use the same discrete-uniform source distribution on a square count grid covering the displayed data range, while Poisson-JUMP uses its own Poisson-based source construction. We compare both count-FM and count-FM-OT against several representative discrete generative baselines: Dirichlet-FM [Stark et al., 2024] and discrete-FM [Gat et al., 2024] from discrete flow matching, D3PM [Austin et al., 2021], tauLDR [Campbell et al., 2022], and SEDD [Lou et al., 2024] from discrete diffusion-style modeling, and Poisson-JUMP, a count-specific jump model. Most of these baselines are based on categorical-state parameterizations. Poisson-JUMP instead works directly with counts, but unlike count-FM it does not learn an explicit conditional bridge with local birth and death rates. For count-FM, we use a small time-varying MLP backbone with hidden width 32, with shared hidden layers and separate output channels for the birth and death rates. For the competing methods, we use standard or matched-capacity architectures.

We evaluate sample quality using the 2-Wasserstein distance and ${\text{MMD}}_{\text{RBF}}^{2}$, the squared maximum mean discrepancy with a Gaussian RBF kernel [Gretton et al., 2012]. We repeat the evaluation over five random seeds and report the mean and standard deviation in Table 1. Our count-FM achieves the best mean $W_{2}$ and ${\text{MMD}}_{\text{RBF}}^{2}$, with count-FM-OT performing comparably. Notably, this performance is achieved with substantially fewer trainable parameters (Table 1).

Figure 3 in Appendix B shows representative intermediate samples along each model’s native sampling trajectory. Figure 4 further compares the marginal bridges under a common progress variable, with Poisson-JUMP excluded because no analogous bridge is available. Under this normalization, both count-FM variants evolve smoothly in count space, while count-FM-OT follows a visibly straighter transition path. In contrast, the categorical-state diffusion- and flow-based baselines move most mass toward the target early along the common progress scale, indicating a more abrupt transition in count space. This geometric advantage is also reflected in sampling efficiency. Appendix Figure 5 shows that count-FM-OT generally reaches a given $W_{2}$ or ${\text{MMD}}_{\text{RBF}}^{2}$ level with fewer NFEs or less runtime than the independently coupled version.

## 4 Applications

### 4.1 Single-cell RNA-seq generation and transport

We study the Dentate Gyrus scRNA-seq dataset [Hochgerner et al., 2018], which contains 2,930 cells and 13,913 genes, together with cell-type and developmental-age annotations spanning multiple lineages. The two developmental time points used in our transport experiment are postnatal day 12 (P12, 1,124 cells) and postnatal day 35 (P35, 1,806 cells). We consider two tasks: unconditional generation from the marginal distribution and transport from P12 to P35 along development.

#### 4.1.1 Unconditional generation

For unconditional generation, each model is trained to generate full gene-expression count vectors from the marginal cell distribution. We use a random 80/20 train-test split over all 2,930 cells, giving 2,344 training cells and 586 held-out test cells. We compare count-FM and count-FM-OT with representative baselines for single-cell generation, including scVI [Lopez et al., 2018] as a latent-variable baseline, scDiffusion [Luo et al., 2024] and scLDM [Palla et al., 2025] as latent diffusion models, and DCM [Bhattacharya et al., 2026] and CFGen [Palma et al., 2025] as additional recent generative baselines for scRNA count data. For both scRNA experiments, count-FM-(OT) uses a shared transformer backbone with hidden dimension 256, depth 8, and 8 attention heads, with separate output channels for the birth and death rates. The competing methods use their implementations with comparable size settings for a fair comparison.

We evaluate held-out sample quality using $W_{2}$ and ${\text{MMD}}_{\text{RBF}}^{2}$ computed in a PCA feature space built from the top 2,000 variable genes after normalization and log transformation, with the number of PCs chosen to explain 90% of the variance. The results are summarized in Table 2. Both count-FM variants outperform the competing methods, with count-FM achieving the best performance and count-FM-OT performing comparably. As in the simulation experiment (Section 3), OT endpoint coupling improves sampling efficiency at lower budgets, with count-FM-OT reaching comparable quality using fewer NFEs or less runtime, while the gap becomes smaller at larger budgets (Appendix Figure 5). Among the models with available parameter counts, count-FM and count-FM-OT achieve strong held-out sample quality with a substantially more efficient parameterization.

#### 4.1.2 Transport from P12 to P35

We next study transport between postnatal days 12 (P12) and 35 (P35) in the Dentate Gyrus dataset. A key advantage of count-FM is that it defines transport directly in count space, so the intermediate states remain count-valued samples and are therefore interpretable as meaningful transitional distributions. This is especially useful in developmental settings, where one would like to inspect not only the endpoints but also the full transition path. Related single-cell methods have likewise emphasized reconstructing developmental trajectories and fate structure, for example through optimal transport and fate-mapping frameworks [Schiebinger et al., 2019, Lange et al., 2022].

The Dentate Gyrus data have a dominant developmental trajectory along the granule-cell lineage, while several mature populations form comparatively stable side lineages. In particular, prior analyses [Cui et al., 2024] of this dataset identify the main progression from neuroblast cells to granule immature cells and then to granule mature cells, whereas mature side populations evolve separately rather than along the main granule trajectory [Hochgerner et al., 2018]. To avoid source-target pairings driven only by geometric proximity, we transport P12 count profiles to P35 count profiles using lineage-restricted OT couplings. Specifically, OT matching is performed only within lineage-consistent source-target groups, so the learned transport respects known developmental structure and avoids biologically implausible transitions across incompatible cell states. This restriction encourages straighter, lower-curvature paths that are easier to interpret biologically. We use separate 80/20 train-test splits for each subset, stratified by cell cluster to preserve cluster composition.

**Figure 1:**
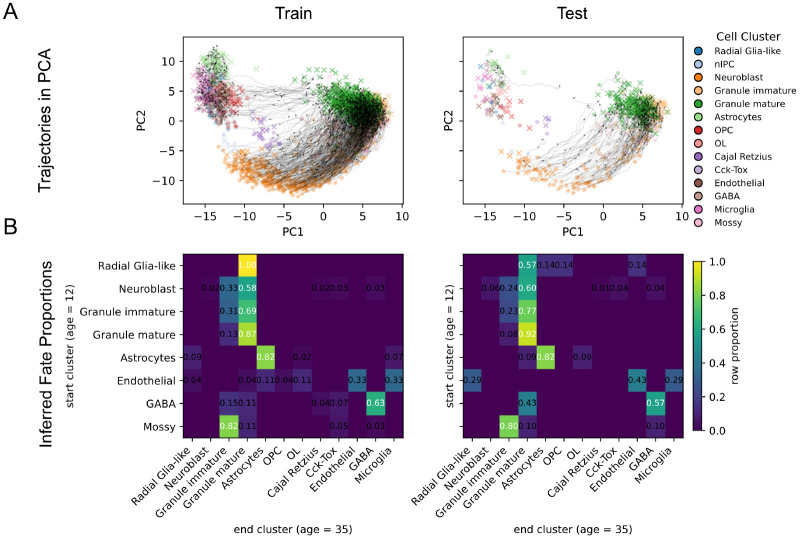
Transport from P12 to P35. **A.** Generated transport trajectories in PCA space for the training and test splits. Circles denote the P12 source cells and crosses denote the P35 target cells, with colors indicating cell cluster identity. Black curves show representative generated trajectories, which smoothly connect P12 to P35. **B.** Inferred fate proportions under P12-to-P35 transport. Rows correspond to source clusters at P12 and columns to inferred endpoint clusters at P35. Entries are row-normalized proportions for the training and test splits.

Figure 1 summarizes the P12-to-P35 transport results. In panel A, generated trajectories move smoothly from the P12 cells toward the P35 cells, with colors indicating cell lineage. In panel B, the inferred terminal states are largely lineage-consistent. Neuroblast and granule immature cells predominantly map to granule immature or granule mature states, while mature side populations such as astrocytes, endothelial, and GABA cells mostly remain within their own lineages. Appendix Figures 6 and 7 further show that the transition unfolds progressively over time on both the training and test sets. Together, these results illustrate how count-FM can be used to model structured developmental transport directly in count space.

### 4.2 Conditional generation on neural spike counts

**Figure 2:**
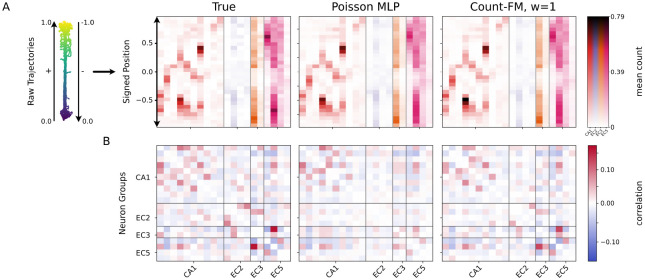
Conditional generation on the hc-3 linear-track session. Summaries are estimated from 100 generated samples per held-out covariate. **A.** Signed position is constructed from linearized position and running direction. Mean-response heatmaps show bin-wise mean counts for the true held-out data, Poisson MLP, and count-FM with w=1. **B.** Population correlation matrices for the same active-neuron set. Count-FM better preserves cross-neuron dependence, while Poisson MLP underestimates population correlations.

**Figure 3:**
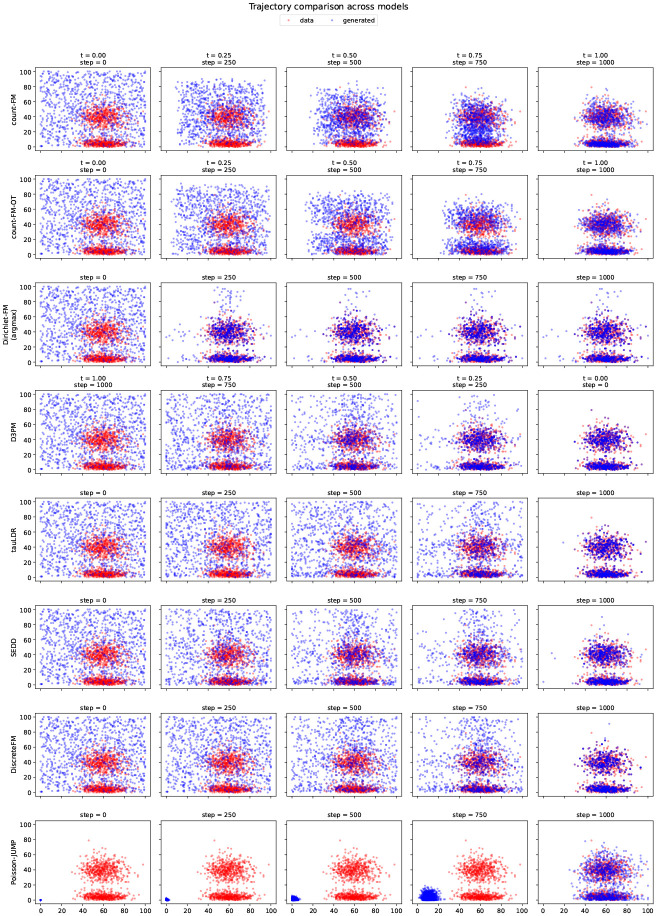
Representative intermediate samples from the native sampling trajectories of different models in the simulation. Red points show target samples and blue points show generated samples. Columns correspond to increasing native sampling time or sampling step within each method’s own generation procedure.

**Figure 4:**
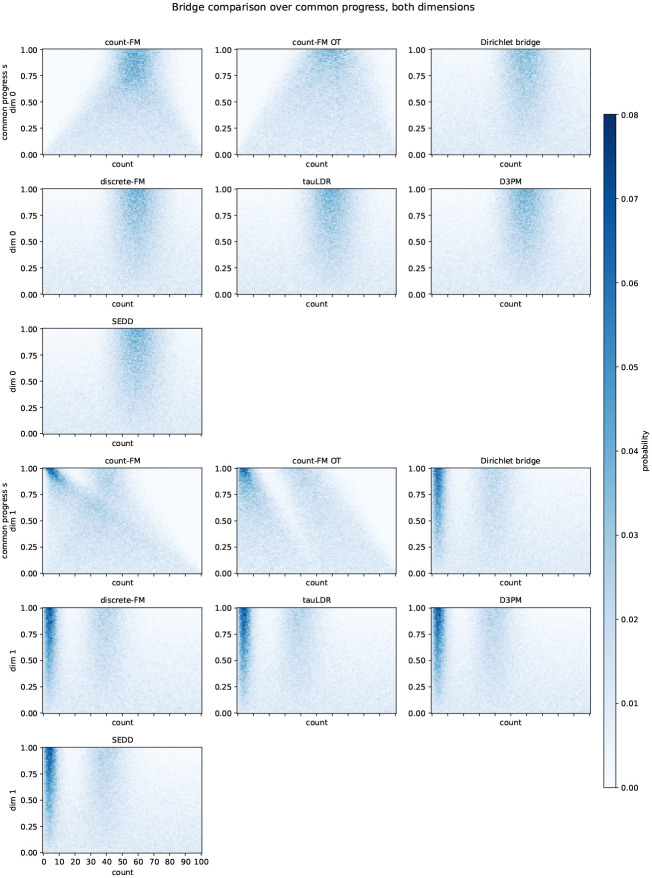
Marginal bridge distributions for both coordinates. They are shown under a common progress variable s. Intermediate marginals are estimated by Monte Carlo sampling from each model’s bridge or forward noising law after mapping to the common progress scale. count-FM evolves gradually across s, while categorical-state baselines become target-like early. count-FM-OT uses the same bridge as count-FM but with OT-coupled endpoints, yielding a visibly straighter transition path.

We next study conditional generation of neural spike counts from a large collection of multi-region hippocampal and entorhinal cortex recordings in behaving Long-Evans rats. In our experiment, we focus on one linear-track session (ec013.719) from the CRCNS hc-3 dataset [Mizuseki et al., 2013, 2014], retaining 62 simultaneously recorded units from CA1, EC2, EC3, and EC5, yielding 29,239 observations after preprocessing. We use an 80/20 train-test split, giving 23,391 training observations and 5,848 held-out observations. The covariates are linearized position and running direction, combined into a single signed position (Figure 2A).

Here, in contrast to generative benchmarking (simulation, Section 3) and unconditional generation (scRNA-seq, Section 4.1.1), we focus on conditional count modeling versus standard regression baselines. Specifically, we compare count-FM with guidance scales w∈{0,1,2} against two reference models, a deterministic MLP regressor, which predicts only conditional means, and a Poisson MLP baseline, which models count noise but has limited ability to capture complex correlation structure. Count-FM instead models a complicated joint conditional count distribution and supports classifier-free guidance (CFG), which controls the strength of conditional information without training an auxiliary classifier, as described in Section 2.4. For a fair comparison, all models use comparable width-128 three-layer MLP backbones with SELU activations.

Figure 2 shows representative results for location-modulated neurons from these regions. Many recorded neurons in hippocampal and entorhinal regions are silent or only weakly location-specific, including many interneuron-like units [Thompson and Best, 1989, Epsztein et al., 2011, Hangya et al., 2010], so we display a subset of neurons from each region to make the spatial response pattern visible. In the mean-response heatmaps (Figure 2A and Appendix Figure 8), all models recover the main spatial response patterns. These response patterns are well known in classic findings that hippocampal neurons encode position and can be modulated by running direction [McNaughton et al., 1983, Markus et al., 1995]. However, the models differ more clearly in their population dependence structure (Figure 2B). Count-FM with w=1 better preserves the observed cross-neuron correlation pattern, whereas the Poisson MLP underestimates the dependence structure.

We further examine the effect of guidance scale in Appendix D.2. For mean responses (Appendix Figure 8), count-FM with w=0 lacks conditional information and only captures marginal firing-rate differences, while w=2 sharpens location-specific responses but reduces calibration to the true data. For population correlation structure (Appendix Figure 9), the MLP mean regressor is excluded because it is not a generative count model. Count-FM with w=0 still captures some marginal population dependence, Poisson MLP substantially underestimates correlations, and w=2 amplifies dependence at the cost of calibration, consistent with the discussion in Section 2.4.

To quantify these qualitative observations, we report several held-out metrics in Table 3. For a signed-position bin b and neuron j, let $\mu_{bj}$ denote the held-out mean count and ${\overset{ˆ}{\mu }}_{bj}$ the model mean, estimated from 50 generated samples for generative models. We evaluate ${\text{RMSE}}_{\mu }=\sqrt{\frac{\sum_{b}n_{b}\sum_{j}{\left({\overset{ˆ}{\mu }}_{bj}-\mu_{bj}\right)}^{2}}{\sum_{b}n_{b}d}}$ and analogously compute ${\text{RMSE}}_{\text{var}}$ and ${\text{RMSE}}_{0}$ using the bin-wise variance and zero fraction. To evaluate correlation structure, we compute the off-diagonal covariance matrix $C_{b}$ of active neurons within each bin and calculate ${\text{Cov}}_{\text{F}}=\frac{\sum_{b}n_{b}{\left\|{\overset{ˆ}{C}}_{b}-C_{b}\right\|}_{F}}{\sum_{b}n_{b}}$, where ${\overset{ˆ}{C}}_{b}$ is the corresponding off-diagonal covariance matrix computed from generated samples in bin b. We also summarize place-field sharpness by the raw contrast ${\text{contrast}}_{j}=\frac{{\text{max}}_{b}{\overset{ˆ}{\mu }}_{bj}-{\text{median}}_{b}\left({\overset{ˆ}{\mu }}_{bj}\right)}{{\text{mean}}_{b}\left({\overset{ˆ}{\mu }}_{bj}\right)}$, averaged over active neurons. Larger contrast indicates stronger peak-to-background tuning.

Table 3 summarizes results over 5 replicated runs, each using a different random seed for train-test splitting and model retraining. Count-FM with w=1 gives the strongest overall generative performance, with the lowest ${\text{RMSE}}_{\mu },{\text{RMSE}}_{\text{var}},{\text{RMSE}}_{0}$, and ${\text{Cov}}_{\text{F}}$, indicating best fit to mean firing rate, sparsity, and correlation structure. The Poisson MLP baseline remains competitive for the mean response, but its variance, zero-fraction, and covariance errors are substantially larger. The unconditional model (w=0) has no spatial specificity. Increasing guidance to w=2 produces the sharpest place fields, but worsens most fidelity metrics, indicating over-sharpening. Overall, w=1 gives the best balance between spatial tuning and distributional calibration.

In a separate dataset, we also evaluate conditional generation on piriform cortex odor-response spike trains in Appendix D.3, where count-FM shows a similar guidance tradeoff. The mean MLP and Poisson MLP baselines recover only coarse response structure, with overly smooth or diffuse responses. In contrast, count-FM better captures odor- and respiration-dependent response patterns. Moderate guidance (e.g., w=1) gives better calibration, while stronger guidance (e.g., w=2) sharpens the pattern but can over-amplify spike counts.

## 5 Discussion

In this work, we introduced count-FM, a flow-matching framework for count data based on local birth-death dynamics. By modeling transport through local births and deaths directly in count space, count-FM respects the underlying count geometry while avoiding the large categorical-state output parameterizations used by many existing discrete generative models. In simulation and in applications to scRNA-seq and neural spike trains, count-FM achieved high sample quality with fewer parameters, while its intermediate paths remain in count space and are therefore interpretable as source-to-target transformations.

Although count-FM performed well across our experiments, the model still has several limitations and natural directions for improvement. First, the conditional binomial bridge is deliberately simple, but it may not be stochastic enough to adequately explore plausible intermediate paths. This may increase extrapolation error and make the learned source-to-target transition overly rigid. A natural extension is to replace it with a more flexible stochastic bridge, for example a beta-binomial variant, or more generally a latent stochastic path construction in the spirit of stochastic interpolants [Albergo and Vanden-Eijnden, 2023, Albergo et al., 2025] and GP-CFM [Wei and Ma, 2025]. Second, our current sampler uses a first-order local one-step discretization, which may not be sufficiently accurate numerically. More accurate bridge-aware simulation schemes [Hobolth and Stone, 2009] and inference-time correction ideas related to Feynman-Kac methods [Skreta et al., 2025] may therefore improve fidelity. Third, the current sampling scheme requires many small steps. Future work might consider accelerated samplers that skip intermediate steps [Frans et al., 2025] or more efficient transition operators through inner-flow sampling [Holderrieth et al., 2026] or flow-map learning [Boffi et al., 2025, Potaptchik et al., 2026]. Because the conditional binomial bridge is analytically simple, such operators may also be easier to derive in closed form.

In summary, we introduced count-FM, a flow-matching framework for count data based on local birth-death dynamics. Across simulation and applications to biomedical data, including scRNA-seq and neural spike trains, count-FM achieved strong sample quality with fewer parameters than competing categorical-state approaches, while preserving count-valued intermediate paths. These results suggest that count-FM provides an effective framework for modeling high-dimensional count data by enabling generation and transport directly in the count space.

## Figures and Tables

**Table 1:** Performance comparison on the two-dimensional simulation. Results are reported as mean ± standard deviation over 5 repeated runs. All models except Poisson-JUMP use the same discrete-uniform source distribution for a fair comparison. Model names include the number of trainable parameters in parentheses. count-FM achieves the best mean $W_{2}$ and ${\text{MMD}}_{\text{RBF}}^{2}$, with count-FM-OT performing comparably, despite both using far fewer parameters than the categorical-state baselines.

Model (params)	$W_{2}↓$	${\text{MMD}}_{\text{RBF}}^{2}↓$
count-FM (2,372)	**2.879** ± **0.380**	**0.0001** ± **0.0007**
count-FM-OT (2,372)	2.971 ± 0.472	0.0002 ± 0.0006
D3PM (15,306)	3.283 ± 0.510	0.0006 ± 0.0008
discrete-FM (14,250)	3.360 ± 0.762	0.0009 ± 0.0015
tauLDR (15,306)	3.733 ± 0.732	0.0008 ± 0.0010
SEDD (15,306)	4.426 ± 0.811	0.0026 ± 0.0016
Poisson-JUMP (67,330)	4.581 ± 0.595	0.0038 ± 0.0013
Dirichlet-FM (15,306)	6.191 ± 0.677	0.0036 ± 0.0018

**Table 2:** Performance comparison across generative models on the Dentate Gyrus dataset for unconditional generation. Results are reported as mean ± standard deviation over 5 repeated runs. Model names include the number of trainable parameters in parentheses when available. count-FM achieves the best performance on both $W_{2}$ and ${\text{MMD}}_{\text{RBF}}^{2}$, while count-FM-OT performs comparably.

Model (params)	$W_{2}↓$	${\text{MMD}}_{\text{RBF}}^{2}↓$
count-FM (9,857,792)	**20.456** ± **0.055**	**0.0185** ± **0.0005**
count-FM-OT (9,857,792)	20.553 ± 0.054	0.0195 ± 0.0007
scLDM	20.739 ± 0.018	0.0202 ± 0.0003
scDiffusion (34,704,897)	20.545 ± 0.051	0.0225 ± 0.0004
scVI	20.687 ± 0.042	0.0229 ± 0.0002
DCM (39,848,665)	23.566 ± 0.010	0.0296 ± 0.0000
CFGen (29,165,380)	22.226 ± 0.106	0.0361 ± 0.0008

**Table 3:** Conditional generation on the hc-3 linear-track session. Results are mean ± standard deviation across 5 random seeds. count-FM with w=1 gives the best performance on mean firing rate $\left({\text{RMSE}}_{\mu }\right)$, sparsity $\left({\text{RMSE}}_{0}\right)$, and covariance structure (${\text{RMSE}}_{\text{var}}$ and $\left.{\text{Cov}}_{\text{F}}\right)$, while w=2 gives the largest contrast but worsens calibration.

Model	${\text{RMSE}}_{\mu }↓$	${\text{RMSE}}_{\text{var}}↓$	${\text{RMSE}}_{0}↓$	${\text{Cov}}_{\text{F}}↓$	Contrast
MLP mean	0.026 ± 0.002	–	–	–	3.087 ± 0.034
Poisson MLP	0.027 ± 0.002	0.115 ± 0.003	0.027 ± 0.001	0.564 ± 0.012	3.134 ± 0.102
count-FM, w=0	0.070 ± 0.002	0.110 ± 0.005	0.043 ± 0.000	0.451 ± 0.021	0.109 ± 0.007
count-FM, w=1	**0.026** ± **0.002**	**0.048** ± **0.005**	**0.016** ± **0.001**	**0.344** ± **0.017**	3.209 ± 0.086
count-FM, w=2	0.061 ± 0.002	0.094 ± 0.007	0.036 ± 0.001	0.451 ± 0.020	**4.521** ± **0.076**

**Figure 5:**
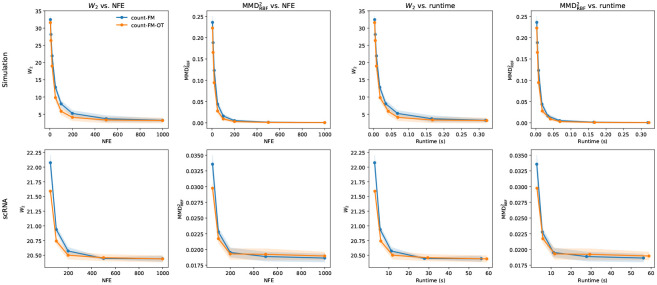
Sampling efficiency comparison between count-FM and count-FM-OT in the simulation and scRNA experiments. We plot $W_{2}$ and ${\text{MMD}}_{\text{RBF}}^{2}$ against the number of function evaluations (NFE) and wall-clock runtime. In the simulation, count-FM-OT reaches a given quality level with smaller computational budget. On the scRNA task, the same trend is present but weaker, and the difference becomes small at larger budgets.

**Figure 6:**
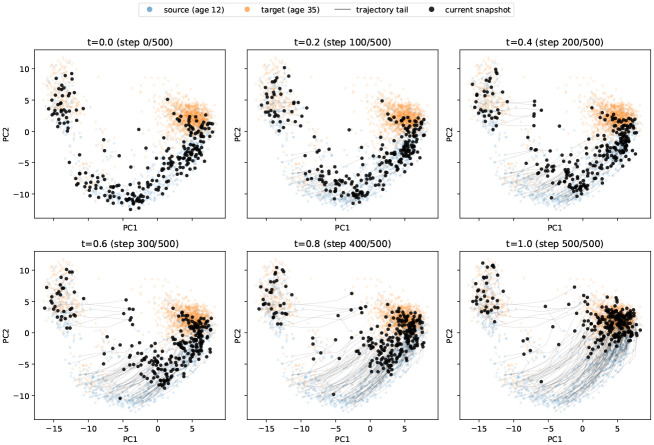
Transition snapshots for training. As time increases, generated cells move progressively from the P12 source manifold toward the P35 target manifold in PCA space. Light colored points show the source and target references, gray curves show trajectory tails, and black points show the current generated snapshot.

**Figure 7:**
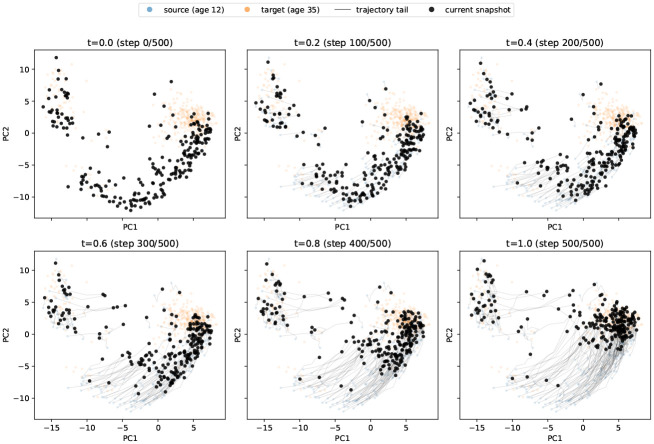
Transition snapshots for testing. The same progressive transport pattern is observed on held-out cells, with trajectories moving smoothly from the P12 manifold toward the P35 manifold.

**Figure 8:**
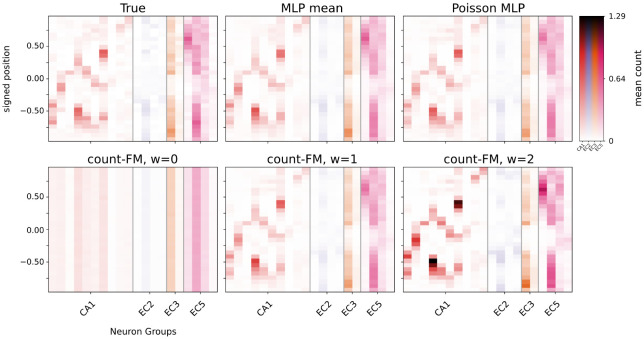
Mean-response comparison for the hc-3 conditional-generation task. Each panel shows bin-wise mean counts across signed position, with neurons grouped by region. For generative models, summaries are estimated from 100 generated samples per held-out covariate. The MLP mean regressor, Poisson MLP, and count-FM with w=1 recover the broad spatial response pattern. Count-FM with w=0 lacks spatial specificity, while w=2 sharpens location-specific responses but reduces calibration.

**Figure 9:**
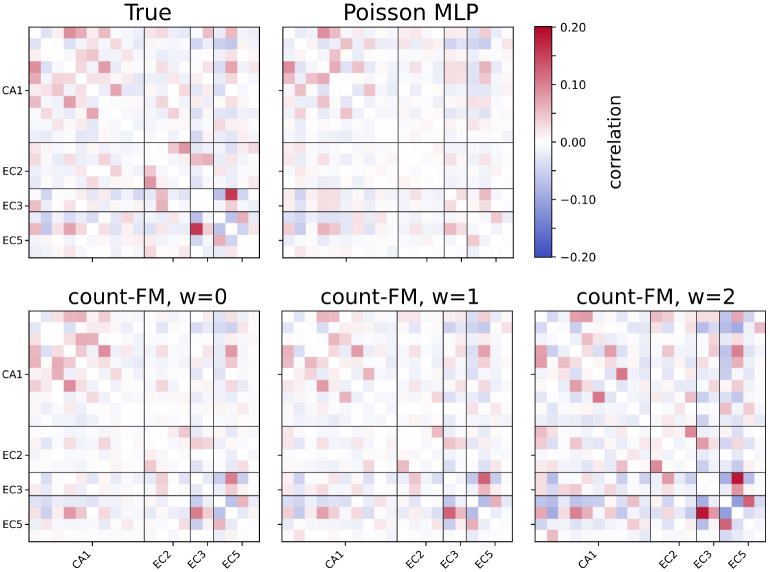
Population correlation comparison for the hc-3 conditional-generation task. Panels show correlation matrices for the active-neuron set. These are estimated from 100 generated samples per held-out covariate. Count-FM with w=1 most closely matches the true dependence structure, while the Poisson MLP underestimates population correlations. Count-FM with w=0 still captures some marginal dependence structure, and stronger guidance (w=2) amplifies the correlation pattern but is less well calibrated.

**Figure 10:**
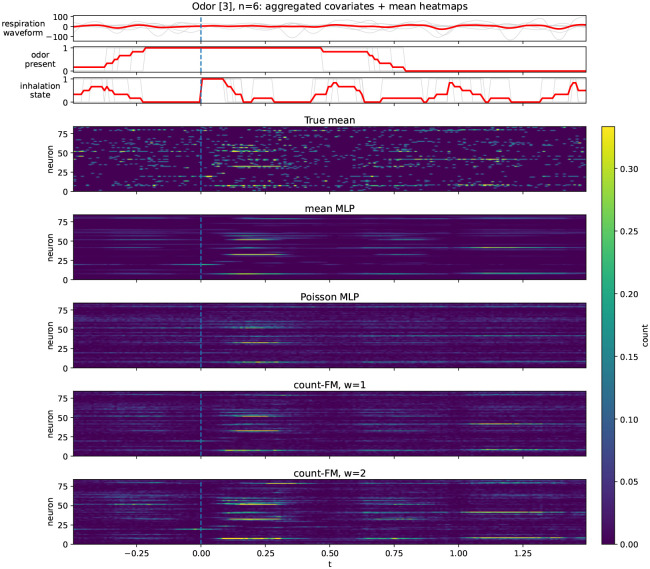
Conditional generation on held-out odor-3 trials. Upper panel shows conditioning covariates over time, with gray traces for individual held-out trials and red traces for trial averages. Lower panel shows neuron-by-time mean responses for the held-out data, the MLP mean regressor, Poisson MLP, and count-FM with w=1 and w=2. The MLP mean regressor is overly smooth and Poisson MLP remains diffuse, while count-FM better recovers localized odor- and respiration-dependent responses. Increasing guidance to w=2 sharpens the response pattern but over-amplifies spike counts, reducing amplitude calibration.

**Figure 11:**
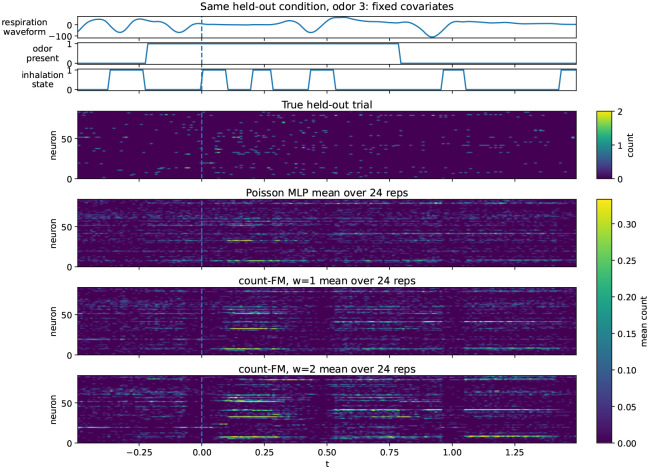
Generated responses under the same held-out covariate trajectory. We compare the true held-out trial with the mean response from the Poisson MLP regressor over 24 repetitions, and with the count-FM mean responses over 24 repetitions at w=1 and w=2. Compared with the Poisson MLP regressor, count-FM produces a more structured condition-aligned response. Increasing guidance from w=1 to w=2 sharpens the shape further, but also inflates the response amplitude.

## A Method details and derivations

This appendix provides derivations and technical details for the construction in Section 2. We first derive the conditional bridge rates, then derive the local rate-matching objective and show how the local KL terms integrate to the path-space KL objective. We also describe the discretization used for sampling in Section 2.2.

### A.1 Derivation of conditional bridge rates

This subsection derives the conditional birth and death rates used in Section 2.1. We work in one dimension and suppress the coordinate index i. Let $x_{0},x_{1}\in N_{0}$ and define the bridge

$$X_{t}=x_{0}+\text{sgn}\left(x_{1}-x_{0}\right)B_{t},\;B_{t}\sim \text{Binomial}\left(\left|x_{1}-x_{0}\right|,t\right).$$

For fixed endpoints ${(x}_{0},x_{1})$, let

$$p_{t}\left(x|x_{0},x_{1}\right)=P\left(X_{t}=x|x_{0},x_{1}\right)$$

denote the one-dimensional conditional bridge pmf. Then mass preservation is

(5)
$$\partial_{t}p_{t}\left(x|x_{0},x_{1}\right)=\lambda_{t}\left(x-1|x_{0},x_{1}\right)p_{t}\left(x-1|x_{0},x_{1}\right)+\mu_{t}\left(x+1|x_{0},x_{1}\right)p_{t}\left(x+1|x_{0},x_{1}\right)\;-\left(\lambda_{t}\left(x|x_{0},x_{1}\right)+\mu_{t}\left(x|x_{0},x_{1}\right)\right)p_{t}\left(x|x_{0},x_{1}\right).$$

This is the one-dimensional special case of the Kolmogorov forward equation (KFE) for CTMCs [Campbell et al., 2022].

**Case 1:** $x_{1}\geq x_{0}$. In this case, the bridge is increasing, with

ptx∣x0,x1=x1-x0x-x0tx-x0(1-t)x1-x,x∈x0,…,x1,

and $\mu_{t}\left(x|x_{0},x_{1}\right)=0$ for 0≤t≤1. Hence mass preservation reduces to

(6)
$$\partial_{t}p_{t}\left(x|x_{0},x_{1}\right)=\lambda_{t}\left(x-1|x_{0},x_{1}\right)p_{t}\left(x-1|x_{0},x_{1}\right)-\lambda_{t}\left(x|x_{0},x_{1}\right)p_{t}\left(x|x_{0},x_{1}\right).$$

Differentiating the binomial pmf gives

$$\partial_{t}p_{t}\left(x|x_{0},x_{1}\right)=\left(\frac{x-x_{0}}{t}-\frac{x_{1}-x}{1-t}\right)p_{t}\left(x|x_{0},x_{1}\right),$$

and

$$\frac{p_{t}\left(x-1|x_{0},x_{1}\right)}{p_{t}\left(x|x_{0},x_{1}\right)}=\frac{x-x_{0}}{x_{1}-x+1}\cdot \frac{1-t}{t}.$$

Let

$$\lambda_{t}\left(x|x_{0},x_{1}\right)=\frac{x_{1}-x}{1-t},$$

then

$$\lambda_{t}\left(x-1|x_{0},x_{1}\right)p_{t}\left(x-1|x_{0},x_{1}\right)=\frac{x-x_{0}}{t}p_{t}\left(x|x_{0},x_{1}\right),$$

and hence (6) holds. Therefore, for $x_{1}\geq x_{0}$,

$$\lambda_{t}\left(x|x_{0},x_{1}\right)=\frac{x_{1}-x}{1-t},\;\mu_{t}\left(x|x_{0},x_{1}\right)=0.$$

**Case 2:** $x_{1}<x_{0}$. In this case, the bridge is decreasing, with

ptx∣x0,x1=x0-x1x0-xtx0-x(1-t)x-x1,x∈x1,…,x0,

and $\lambda_{t}\left(x|x_{0},x_{1}\right)=0$ for 0≤t≤1. Mass preservation reduces to

(7)
$$\partial_{t}p_{t}\left(x|x_{0},x_{1}\right)=\mu_{t}\left(x+1|x_{0},x_{1}\right)p_{t}\left(x+1|x_{0},x_{1}\right)-\mu_{t}\left(x|x_{0},x_{1}\right)p_{t}\left(x|x_{0},x_{1}\right).$$

Applying the same calculation as in Case 1 to the reduced mass-preservation equation (7) gives

$$\mu_{t}\left(x|x_{0},x_{1}\right)=\frac{x-x_{1}}{1-t},\;\lambda_{t}\left(x|x_{0},x_{1}\right)=0.$$

Combining the two cases yields

$$\lambda_{t}\left(x|x_{0},x_{1}\right)=\frac{{\left(x_{1}-x\right)}_{+}}{1-t},\;\mu_{t}\left(x|x_{0},x_{1}\right)=\frac{{\left(x-x_{1}\right)}_{+}}{1-t}.$$

Applying this coordinatewise gives the formula in Section 2.1.

### A.2 Local KL derivation of the rate-matching training objective

This subsection derives the rate-matching training objective (4) in Section 2.1 from the infinitesimal KL divergence between the target and model one-step transition probabilities.

Fix a bridge state x at time t. For a small step size h>0, define the target local transition law $q_{h}\left(\cdot |x,x_{0},x_{1}\right)$ by

$$q_{h}\left(x+e_{i}|x,x_{0},x_{1}\right)=h\;\lambda_{t,i}\left(x|x_{0},x_{1}\right)+o\left(h\right),$$

$$q_{h}\left(x-e_{i}|x,x_{0},x_{1}\right)=h\;\mu_{t,i}\left(x|x_{0},x_{1}\right)+o\left(h\right).$$

and

$$q_{h}\left(x|x,x_{0},x_{1}\right)=1-h\sum_{i=1}^{d}\left(\lambda_{t,i}\left(x|x_{0},x_{1}\right)+\mu_{t,i}\left(x|x_{0},x_{1}\right)\right)+o(h).$$

The model for local transition probability $q_{h}^{\theta }(\cdot |x,t)$ is defined analogously by replacing $\lambda_{t,i}(x|\left.x_{0},x_{1}\right),\mu_{t,i}\left(x|x_{0},x_{1}\right)$ with $\lambda_{\theta ,i}(x,t),\mu_{\theta ,i}(x,t)$.

Let

$$a_{t}\left(x|x_{0},x_{1}\right)=\sum_{i=1}^{d}\left(\lambda_{t,i}\left(x|x_{0},x_{1}\right)+\mu_{t,i}\left(x|x_{0},x_{1}\right)\right),\;a_{t}^{\theta }(x,t)=\sum_{i=1}^{d}\left(\lambda_{\theta ,i}(x,t)+\mu_{\theta ,i}(x,t)\right).$$

We consider the local KL divergence

$$\text{KL}\left(q_{h}\left(\cdot |x,x_{0},x_{1}\right)\|q_{h}^{\theta }(\cdot |x,t)\right).$$

For the no-jump event,

$$q_{h}\left(x|x,x_{0},x_{1}\right)\text{log}\frac{q_{h}\left(x|x,x_{0},x_{1}\right)}{q_{h}^{\theta }\left(x|x,t\right)}=\left(1-h\;a_{t}\left(x|x_{0},x_{1}\right)+o\left(h\right)\right)\text{log}\frac{1-h\;a_{t}\left(x|x_{0},x_{1}\right)+o\left(h\right)}{1-h\;a_{t}^{\theta }\left(x,t\right)+o\left(h\right)}.$$

As h→0, using log(1+u)=u+o(u) as u→0, and noting that $q_{h}\left(x|x,x_{0},x_{1}\right)=1+O(h)$,

$$q_{h}\left(x|x,x_{0},x_{1}\right)\text{log}\frac{q_{h}\left(x|x,x_{0},x_{1}\right)}{q_{h}^{\theta }\left(x|x,t\right)}=h\left(a_{t}^{\theta }\left(x,t\right)-a_{t}\left(x|x_{0},x_{1}\right)\right)+o\left(h\right).$$

For the jump events,

$$q_{h}\left(x+e_{i}|x,x_{0},x_{1}\right)\text{log}\frac{q_{h}\left(x+e_{i}|x,x_{0},x_{1}\right)}{q_{h}^{\theta }\left(x+e_{i}|x,t\right)}=h\;\lambda_{t,i}\left(x|x_{0},x_{1}\right)\text{log}\frac{\lambda_{t,i}\left(x|x_{0},x_{1}\right)}{\lambda_{\theta ,i}\left(x,t\right)}+o\left(h\right),$$

and similarly,

$$q_{h}\left(x-e_{i}|x,x_{0},x_{1}\right)\text{log}\frac{q_{h}\left(x-e_{i}|x,x_{0},x_{1}\right)}{q_{h}^{\theta }\left(x-e_{i}|x,t\right)}=h\;\mu_{t,i}\left(x|x_{0},x_{1}\right)\text{log}\frac{\mu_{t,i}\left(x|x_{0},x_{1}\right)}{\mu_{\theta ,i}(x,t)}+o(h).$$

Summing all contributions gives

$$\text{KL}\left(q_{h}\left(\cdot |x,x_{0},x_{1}\right)\|q_{h}^{\theta }(\cdot |x,t)\right)=h\sum_{i=1}^{d}\left[\lambda_{t,i}\left(x|x_{0},x_{1}\right)\text{log}\frac{\lambda_{t,i}\left(x|x_{0},x_{1}\right)}{\lambda_{\theta ,i}\left(x,t\right)}-\lambda_{t,i}\left(x|x_{0},x_{1}\right)+\lambda_{\theta ,i}(x,t)\right]\;+h\sum_{i=1}^{d}\left[\mu_{t,i}\left(x|x_{0},x_{1}\right)\text{log}\frac{\mu_{t,i}\left(x|x_{0},x_{1}\right)}{\mu_{\theta ,i}(x,t)}-\mu_{t,i}\left(x|x_{0},x_{1}\right)+\mu_{\theta ,i}(x,t)\right]+o\left(h\right).$$

Dividing by h and letting h→0 yields

$$\underset{h\to 0}{\text{lim}}\frac{1}{h}\text{KL}\left(q_{h}\left(\cdot |x,x_{0},x_{1}\right)\|q_{h}^{\theta }(\cdot |x,t)\right)=\sum_{i=1}^{d}D_{\text{GKL}}\left(\lambda_{t,i}\left(x|x_{0},x_{1}\right),\lambda_{\theta ,i}(x,t)\right)+\sum_{i=1}^{d}D_{\text{GKL}}\left(\mu_{t,i}\left(x|x_{0},x_{1}\right),\mu_{\theta ,i}(x,t)\right),$$

where $D_{\text{GKL}}$ denotes the generalized KL divergence

$$D_{\text{GKL}}\left(u,v\right)=u\;\text{log}\frac{u}{v}-u+v.$$

Up to terms independent of θ, this is exactly

$$\sum_{i=1}^{d}\left[\lambda_{\theta ,i}(x,t)-\lambda_{t,i}\left(x|x_{0},x_{1}\right)\;\text{log}\;\lambda_{\theta ,i}(x,t)\right]+\sum_{i=1}^{d}\left[\mu_{\theta ,i}(x,t)-\mu_{t,i}\left(x|x_{0},x_{1}\right)\;\text{log}\;\mu_{\theta ,i}(x,t)\right].$$

Taking expectation over $\left(x_{0},x_{1}\right),t$, and x gives the practical rate-matching objective in (4), up to an additive constant independent of θ.

### A.3 Path-space KL interpretation of the training objective

This subsection integrates the local KL calculation in Appendix A.2 over time to obtain the path-space KL objective in (3).

Let $X_{\left(t,1\right]}≔\left\{X_{s}:t<s\leq 1\right\}$ and define

$$K_{\theta }\left(t\right)≔E_{\begin{array}{c} \left(x_{0},x_{1}\right)\sim \pi ,x\sim p_{t}\left(\cdot |x_{0},x_{1}\right) \end{array}}\left[\text{KL}\left(p\left(X_{\left(t,1\right]}|X_{t}=x,x_{0},x_{1}\right)\|p_{\theta }\left(X_{\left(t,1\right]}|X_{t}=x\right)\right)\right].$$

For 0<h≤1-t,

$$p\left(X_{\left(t,1\right]}|X_{t}=x,x_{0},x_{1}\right)=q_{h}\left(X_{t+h}|x,x_{0},x_{1},t\right)p\left(X_{\left(t+h,1\right]}|X_{t+h},x_{0},x_{1}\right),$$

$$p_{\theta }\left(X_{\left(t,1\right]}|X_{t}=x\right)=q_{h}^{\theta }\left(X_{t+h}|x,t\right)p_{\theta }\left(X_{\left(t+h,1\right]}|X_{t+h}\right).$$

Hence, by the chain rule for KL,

$$\text{KL}\left(p\left(X_{\left(t,1\right]}|X_{t}=x,x_{0},x_{1}\right)\|p_{\theta }\left(X_{\left(t,1\right]}|X_{t}=x\right)\right)=\text{KL}\left(q_{h}\left(\cdot |x,x_{0},x_{1},t\right)\|q_{h}^{\theta }(\cdot |x,t)\right)\;+E_{x^{\prime }\sim q_{h}\left(\cdot |x,x_{0},x_{1},t\right)}\left[\text{KL}\left(p\left(X_{\left(t+h,1\right]}|X_{t+h}=x^{\prime },x_{0},x_{1}\right)\|p_{\theta }\left(X_{\left(t+h,1\right]}|X_{t+h}=x^{\prime }\right)\right)\right].$$

Therefore,

$$K_{\theta }\left(t\right)-K_{\theta }\left(t+h\right)=E_{\begin{array}{c} \underset{x\sim p_{t}\left(\cdot |x_{0},x_{1}\right)}{\left(x_{0},x_{1}\right)\sim \pi ,} \end{array}}\left[\text{KL}\left(q_{h}\left(\cdot |x,x_{0},x_{1},t\right)\|q_{h}^{\theta }(\cdot |x,t)\right)\right].$$

Dividing by h and letting h→0,

$$-\frac{d}{dt}K_{\theta }(t)=E_{\begin{array}{c} \underset{x\sim p_{t}\left(\cdot |x_{0},x_{1}\right)}{\left(x_{0},x_{1}\right)\sim \pi ,} \end{array}}\left[\underset{h\to 0}{\text{lim}}\frac{1}{h}\text{KL}\left(q_{h}\left(\cdot |x,x_{0},x_{1},t\right)\|q_{h}^{\theta }(\cdot |x,t)\right)\right].$$

By Appendix A.2, for each fixed t, the local rate-matching loss equals $-\frac{d}{dt}K_{\theta }(t)$ up to an additive constant $c_{t}$ independent of θ. Hence, ${ℒ}_{\text{train}}(\theta )=-\int_{0}^{1}K_{\theta }^{\prime }(t)dt+c=K_{\theta }(0)-K_{\theta }(1)+c$, where $c=\int_{0}^{1}c_{t}dt$ is a constant independent of θ. Since $K_{\theta }(1)=0$ as $X_{\left(1,1\right]}=\emptyset$,

$${ℒ}_{\text{train}}(\theta )=K_{\theta }(0)+c.$$

Evaluating $K_{\theta }(0)$ gives the path-space KL objective in (3):

$${ℒ}_{\text{train}}(\theta )=E_{\left(x_{0},x_{1}\right)\sim \pi }\left[\text{KL}\left(p\left(X_{\left(0,1\right]}|X_{0}=x_{0},X_{1}=x_{1}\right)\|p_{\theta }\left(X_{\left(0,1\right]}|X_{0}=x_{0}\right)\right)\right]+c.$$

## B Simulation

Unless otherwise stated, all experiments were run on a single NVIDIA RTX 4090 GPU with 24GB memory.

Figure 3 visualizes representative intermediate samples along each model’s native sampling trajectory in the simulation. Since different methods use different native time parameterizations and different sampling mechanisms, this figure is qualitative and is intended only to show how generated samples evolve under each fitted model’s own dynamics.

To compare intermediate bridge behavior across models, we construct, for each method and each coordinate, an empirical family of one-dimensional intermediate marginals indexed by a common progress variable s∈[0,1]. For a fixed value of s, we repeatedly sample endpoint pairs $\left(x_{0}^{\left(m\right)},x_{1}^{\left(m\right)}\right)$, generate an intermediate state $X_{s}^{\left(m\right)}$ using the corresponding model-specific bridge or forward noising law, and estimate the marginal pmf by

$${\hat{p}}_{s}^{\left(i\right)}\left(z\right)=\frac{1}{M}\sum_{m=1}^{M}1\left\{X_{s}^{\left(i,m\right)}=z\right\},\;z\in \left\{0,1,\ldots ,C_{\text{max}}\right\}.$$

The heatmaps in Figure 4 plot ${\hat{p}}_{s}^{\left(i\right)}(z)$ as a function of count value z and common progress s.

For count-FM, the conditional bridge is

$$X_{t}^{\left(i\right)}=x_{0}^{\left(i\right)}+\text{sgn}\left(x_{1}^{\left(i\right)}-x_{0}^{\left(i\right)}\right)B_{t}^{\left(i\right)},\;B_{t}^{\left(i\right)}\sim \text{Binomial}\left(\left|x_{1}^{\left(i\right)}-x_{0}^{\left(i\right)}\right|,t\right),$$

and we set s=t. For count-FM-OT, we use the same conditional bridge, but the endpoints ${(x}_{0},x_{1})$ are first paired using the minibatch OT coupling from Section 2.3. We do not include Poisson-JUMP in Figure 4, because its Poisson-source jump construction does not provide a directly comparable one-parameter bridge or forward noising family under the same common-progress normalization.

For the remaining baselines, since each method uses a different forward process and parameterization, we convert each native time variable to a common progress variable s∈[0,1] by monotone rescaling of a model-specific progress statistic ρ(t),

$$s=\frac{\rho (t)-\rho (0)}{\rho (1)-\rho (0)}.$$

Here ρ(t) measures source-to-target progress along the native path of each method. This places all comparison methods on the same source-to-target scale. Under this normalization, the count-FM bridges evolve gradually across the full range of s, whereas the categorical-state diffusion- and flow-based baselines typically become target-like much earlier, reflecting a sharper transition in count space.

## C OT coupling and sampling efficiency

Figure 5 compares the sampling efficiency of count-FM and count-FM-OT in both the simulation and scRNA experiments. We plot $W_{2}$ and ${\text{MMD}}_{\text{RBF}}^{2}$ against the number of function evaluations (NFE) and wall-clock runtime. In the simulation, count-FM-OT shows a clear efficiency advantage across most computational budgets. On the scRNA task, the advantage is smaller but still visible at lower budgets, while the two methods become very similar as the budget increases.

## D Applications

### D.1 single-cell RNA-seq generation and transport

Appendix Figures 6 and 7 complement the main-text trajectory and fate summaries by visualizing the temporal evolution of the transport process on both the training and test sets.

### D.2 Additional results for hippocampal hc-3

This appendix provides expanded qualitative diagnostics for the hc-3 conditional-generation experiment. For generative models, all plotted summaries are estimated from 100 generated samples per held-out covariate. Appendix Figure 8 shows bin-wise mean responses across signed position for all models, with neurons grouped by brain region. The MLP mean regressor, Poisson MLP, and count-FM with w=1 all recover the broad spatial response pattern. Count-FM with w=0 lacks conditional information and only captures marginal firing-rate differences across neurons, while w=2 sharpens location-specific responses but is less well calibrated to the true mean pattern.

Appendix Figure 9 shows the corresponding population correlation structure using the same neuron set. Since the MLP mean regressor is deterministic and is not a generative count model, it is omitted from this dependence comparison. Count-FM with w=1 most closely matches the true dependence structure, while the Poisson MLP underestimates population correlations. The unconditional model (w=0) still captures some marginal dependence structure, whereas w=2 amplifies the correlation pattern but is less well calibrated. These are consistent with Table 3, where w=1 gives the best overall generative fidelity, whereas w=2 mainly sharpens the response pattern at the cost of calibration.

### D.3 Conditional generation of piriform cortex spike trains

We also evaluate count-FM on conditional generation of multichannel piriform cortex (PCx) spike trains using the public odor-response dataset of Bolding and Franks [2017], Bolding et al. [2020]. The dataset contains processed, spike-sorted extracellular recordings together with simultaneously recorded respiration traces from head-fixed mice. We focus on the PCx recording from session 141208–2 (bank 2), which contains 480 trials. Each trial is aligned to inhalation onset, spikes are binned into 10 ms intervals over the window [−0.5, 1.5] s, and each sample is represented as a time-by-neuron count matrix with 200 time bins and 84 neurons [Miura et al., 2012]. The conditioning variables include time, a respiration-waveform covariate, odor presence, odor identity, and binarized inhalation state. We use a trial-level 80/20 train-test split stratified by odor identity, giving 384 training trials and 96 held-out test trials.

Figure 10 shows mean responses over held-out odor-3 trials. The mean MLP captures the coarse response shape but is smoother than the held-out mean, while the Poisson MLP is a stronger count baseline but remains comparatively diffuse. Count-FM recovers the odor- and respiration-dependent structure better. Increasing guidance from w=1 to w=2 sharpens the aligned response pattern, but also increases its amplitude. After aligning the spiking activity with covariates (upper panel in Figure 10) by time, we can see that the neural activity is strongly correlated with the inhalation state (lower firing rate during inhalation), especially when the odor is present. Figure 11 shows the same guidance tradeoff under a fixed held-out covariate. Here, the mean MLP is not shown because it is not generative.

To quantify what we observed, we calculate different metrics and Table 4 summarizes held-out metrics over 5 replicated runs, each with a new odor-stratified 80/20 train-test split and retraining under different random seeds. Among the count-FM settings, w=1 gives the best overall tradeoff, with the lowest mean RMSE and 95th-percentile error, while remaining close to the Poisson MLP baseline in matched cosine and mean-count ratio. By contrast, w=2 gives the highest matched cosine among count-FM settings, but substantially worse mean RMSE and a larger mean-count ratio, indicating stronger condition alignment at the cost of amplitude distortion.

**Table 4:** Held-out conditional generation quality on the PCx task. Results are mean ± standard deviation over 5 random seeds, each with a new odor-stratified train-test split and model retraining. count-FM with w=1 gives the best overall tradeoff among count-FM settings, while w=2 improves matched cosine but over-amplifies spike counts.

Model	Mean RMSE ↓	Matched cosine ↑	Mean-count ratio	95th-percentile error ↓
MLP mean	1.264 ± 0.248	**0.800** ± **0.041**	0.704 ± 0.100	0.487 ± 0.017
Poisson MLP	1.090 ± 0.071	0.762 ± 0.018	1.073 ± 0.058	0.311 ± 0.069
count-FM, w=0	2.090 ± 0.255	0.407 ± 0.074	1.300 ± 0.429	0.322 ± 0.093
count-FM, w=1	**1.082** ± **0.056**	0.760 ± 0.019	**1.037** ± **0.078**	**0.297** ± **0.071**
count-FM, w=2	1.744 ± 0.342	0.792 ± 0.022	1.427 ± 0.188	0.336 ± 0.029
